# Testing Reported Associations of Gene Variants with Non-Syndromic Orofacial Clefts in the Polish Population

**DOI:** 10.3390/biomedicines12081700

**Published:** 2024-07-31

**Authors:** Alicja Zawiślak, Krzysztof Woźniak, Gianluca Tartaglia, Beata Kawala, Satish Gupta, Anna Znamirowska-Bajowska, Katarzyna Grocholewicz, Jan Lubiński, Anna Jakubowska

**Affiliations:** 1 Department of Maxillofacial Orthopaedics and Orthodontics, Institute of Mother and Child, 01-211 Warsaw, Poland; 2 Department of Interdisciplinary Dentistry, Pomeranian Medical University, 70-111 Szczecin, Poland; katarzyna.grocholewicz@pum.edu.pl; 3 Department of Orthodontics, Pomeranian Medical University, 70-111 Szczecin, Poland; krzysztof.wozniak@pum.edu.pl; 4 Department of Biomedical, Surgical and Dental Sciences, University of Milan, 20100 Milan, Italy; gianluca.tartaglia@unimi.it; 5 Department of Dentofacial Orthopaedics and Orthodontics, Wrocław Medical University, 50-425 Wroclaw, Poland; ortodoncja@umed.wroc.pl (B.K.); aznamirowska.b@gmail.com (A.Z.-B.); 6 Hereditary Cancer Center, Department of Genetics and Pathology, Pomeranian Medical University, 70-111 Szczecin, Poland; satish1482@gmail.com (S.G.); jan.lubinski@pum.edu.pl (J.L.); anna.jakubowska@pum.edu.pl (A.J.); 7 Independent Laboratory of Molecular Biology and Genetic Diagnostics, Pomeranian Medical University, 70-111 Szczecin, Poland

**Keywords:** birth defect, cleft lip, cleft palate, genetic variation, single nucleotide polymorphism, *MYH9*, *MTHFR*, *MAFB*, *SUMO1*

## Abstract

Orofacial clefts (OFCs) are the second most common birth defect worldwide. The etiology of OFCs involves complex interactions between genetics and environment. Advances in genomic technologies have identified gene variants associated with OFCs. This study aimed to investigate whether selected SNPs in the *MYH9*, *MTHFR*, *MAFB*, and *SUMO1* genes influence the occurrence of non-syndromic OFCs in the Polish population. The study included 209 individuals with non-syndromic OFCs and 418 healthy controls. Saliva and umbilical cord blood samples were collected for DNA extraction. Four SNPs in the *MYH9*, *MTHFR*, *MAFB*, and *SUMO1* genes were genotyped using real-time PCR-based TaqMan assays. Statistical analysis was performed using logistic regression to assess the association between SNPs and OFCs. A significant association was found between the rs7078 CC polymorphism and OFCs (OR = 3.22, CI 1.68–6.17, *p* < 0.001). No significant associations were identified for the rs1081131, rs13041247, and rs3769817 polymorphisms. The research indicates that the rs7078 polymorphism significantly influences the occurrence of orofacial cleft palate in the Polish population, whereas the rs3769817, rs1801131, and rs13041247 SNPs do not show such a correlation.

## 1. Introduction

Orofacial clefts (OFCs) are the most common craniofacial congenital deformity worldwide. The frequency of clefts is on average 1 in 700 live births and varies depending on the ethnic background of the population studied [1]. The prevalence rate ranges from 1 in 500 live births in the Asian population to 1 in 2500 in the African population [2].

A cleft defect significantly impacts a child’s development; it not only negatively affects the function of the stomatognathic system, but it also has psychological and socioeconomic impacts [3]. The severity of OFCs depends on the extent of the affected structures. Facial development is an intricate process involving the formation of the palate, occurring from the fourth to the twelfth week of embryonic development. This period is characterized by a sequence of cellular growth, differentiation, migration, and apoptosis [4]. Facial appearance significantly impacts the quality of life of individuals with this malformation, thereby playing a crucial role in their overall wellbeing [5,6].

The causes of cleft defects are very complex and include both environmental and genetic factors. Among the environmental causes are factors resulting from the mother’s lifestyle. In contrast, genetic changes often involve signaling path genes responsible for the formation of craniofacial structures during the early stages of embryogenesis [7].

The classification of OFCs distinguishes between syndromic and non-syndromic clefts, in which the defect is limited solely to the palate itself [8].

With recent advancements in genomic technology, numerous studies have identified various molecular mechanisms involved in the development of both types. Approximately 70% of OFC cases are non-syndromic. Additionally, nearly half of the instances of cleft palate only (CPO) fall into the non-syndromic category [9]. Numerous studies have investigated the identification of genetic loci and candidate genes linked to OFCs using three approaches: linkage studies, the candidate gene approach, and genome-wide association studies (GWAS) [10].

Candidate gene studies are targeted genetic analyses that focus on isolated cases. This approach aims to find a statistically significant association between an allele and a specific phenotype. The candidate gene approach helps identify the causative variant. Selecting candidate genes for study relies on prior knowledge of molecular pathways, focusing on genes known to be involved in facial development and other signaling pathways [10,11].

Mutations in genes involved in craniofacial development are likely candidates for cleft occurrence. In recent years, GWAS and linkage disequilibrium analyses have identified several gene variants—single nucleotide polymorphisms (SNPs)—associated with OFCs [12]. Researchers have confirmed that selected SNPs in genes such as *MYH9, MTHFR, MAFB,* and *SUMO1* play a role in the etiology of OFCs [12,13,14]. One of the most well-studied genes in OFCs encodes the folate metabolism enzyme methylenetetrahydrofolate reductase (*MTHFR*), and in particular it is maternal rather than fetal missense mutations that have been shown to contribute to OFC risk [12]. A huge study involving over 1000 patients with OFCs was conducted in a Chinese Han population, but as a result only two SNPs (namely: rs12107 and rs2269529) in MYH9 were associated with increased expression of *MYH9* and contributed to susceptibility to OFCs [13]. Researchers reported associations with four regions, including two novel loci in V-maf musculoaponeurotic fibrosarcoma oncogene homolog B (*MAFB*), which was confirmed in replication studies. MAFB has been identified in multiple genome scans since [12]. Rs13041247 was identified as the most important SNP and was associated with a decreased risk of the birth defect in a Vietnamese population. The rs13041247 CT, CC, and CT/CC genotypes were linked to decreased susceptibility compared to the homozygous rs13041247 TT wild-type genotype in a Chinese Han population, but only marginal associations were detected for rs13041247 in *MAFB* in a Hispanic dataset, and no association was found in a non-Hispanic white dataset and in Caucasian individuals from Brazil. These findings suggest that racial differences may lead to varying genetic research results [14]. As these findings vary among different ethnic groups, it is important to confirm these results in various populations. Therefore, the aim of our study was to determine whether selected SNPs in the *MYH9* (rs7078), *MTHFR* (rs1801131), *MAFB* (rs13041247), and *SUMO1* (rs3769817) genes influence the occurrence of non-syndromic OFCs in the Polish population.

## 2. Materials and Methods

### 2.1. Study Population

This research study was conducted on an unselected group of individuals with non-syndromic OFCs (*n* = 209) and healthy controls (*n* = 418) with an average age of 14.0 ± 10.2 years. Participants in the study group included patients undergoing orthodontic treatment at the Department of Orthodontics at Pomeranian Medical University in Szczecin and the Department of Dentofacial Orthopedics and Orthodontics at Wroclaw Medical University. In both groups, ancestors of the patients up to the second generation were from the Polish population. The clinical diagnosis of the existing cleft defect, along with the assessment of associated malocclusion, as well as the differential diagnosis for monogenic syndromes associated with cleft lip with or without cleft palate, was based on subjective and objective clinical examinations.

OFCs were categorized according to the World Health Organization’s International Statistical Classification of Diseases and Related Health Problems (ICD-10), specifically under sections Q35–Q37, which address congenital malformations, deformations, and chromosomal abnormalities [15].

The control group consisted of 418 randomly selected patients, whose genetic material derived from umbilical cord blood was stored in the biobank of the Department of Genetics and Pathomorphology at Pomeranian Medical University in Szczecin.

Both the OFC patients and the control group were matched based on age and place of birth to eliminate the influence of external environmental factors, which can modify genetic susceptibility.

### 2.2. Ethical Approval

This study received approval from the Bioethics Committee of the Pomeranian Medical University in Szczecin, adhering to Good Clinical Practice (GCP) guidelines (KB-0012/77/10). Additionally, the oncology biobank project was sanctioned by the Pomeranian Medical University Ethics Committee (BN-001/174/05, dated 11 October 2005). Informed consent was obtained from all participants or their legal guardians before the study commenced.

### 2.3. Samples Preparation and Genotyping

The individuals with OFC provided 2 mL saliva samples using Oragene collection kits (DNA Genotek Inc., Ottawa, ON, Canada). Subjects refrained from consuming solid food for 30 min prior to sample collection. The saliva samples were stored in a dry, light-protected environment at room temperature. DNA isolation was performed using an automated Chemagen set, and the extracted DNA was stored at −20 °C. In the control group, DNA extraction from umbilical cord blood was conducted using the method described by Lahiri et al. [16].

We selected four SNPs located within four genes that have been previously identified as potential candidates for cleft defects, based on research with animal models and human association studies. These SNPs were selected to investigate their association with orofacial clefts in the Polish population, ensuring that the minor allele frequency (MAF) of these SNPs was greater than 0.1. Details of the selected SNPs and genes investigated are presented in Table 1.

Genotyping of rs7078, rs1081131, rs13041247, and rs3769817 was conducted using the real-time PCR-based TaqMan method on the LightCycler 480 II instrument from Roche Diagnostics. Each reaction mixture (total volume 5 μL) comprised 2.5 μL of LightCycler 480 Probes Master Mix (Roche Diagnostics, Basel, Switzerland), 0.0625 μL of each SNP TaqMan Genotyping Assay buffer (Applied Biosystems, Waltham, MA, USA), 1 μL of DNA (25 ng/μL), and 1.4375 μL of deionized water (Roche Diagnostics, Basel, Switzerland). The PCR conditions were previously documented [17]. Additionally, each plate included four negative controls lacking DNA to monitor for potential contamination.

### 2.4. Statistical Analysis

The criterion for statistical significance was established at an alpha level of α = 0.05. For categorical variables, data were summarized by providing counts (n) and percentages (%) for each category.

The association between individual SNP genotypes as exposures and orofacial clefts (OFC) as outcomes was analyzed using the Mantel–Haenszel (M-H) chi-squared test, incorporating sex as a stratifying factor to control for potential confounding effects. In the present case–control study, the association was quantified by measuring the odds of exposure, which were found to be x times greater (or less) in cases compared to controls.

The M–H method, which tests the hypothesis that the combined OR is equal to 1.0, uses a two-sided test without continuity correction. Additionally, the Wald confidence interval (CI) was employed to provide an adjustment for the M–H odds ratio [18].

The M–H adjusted measures of association are considered robust and valid in a clinical context when the association measures across different stratifications exhibit homogeneity. This condition is met when the Woolf test for homogeneity of OR yields a non-significant result [19].

The effect of SNP genotypes on OFC occurrence was evaluated using multivariate analysis with multiple logistic regression. This model used a binomial distribution and a logit link function, with the parameters estimated via maximum likelihood. The accuracy of these estimates was measured by calculating *p*-values and confidence intervals based on the Wald z statistic. Multicollinearity in the regression model was assessed using the Variation Inflation Factor (VIF), with values over 3.0 indicated significant multicollinearity.

Analyses were conducted using the R Statistical language (version 4.3.1; [20]) on Windows 10 pro 64 bit (build 19045), using the packages sjPlot (version 2.8.15; [21]), performance (version 0.10.8; [22], report (version 0.5.7; [23]), gtsummary (version 1.7.2; [24]), epiR (version 2.0.75; [25]), and dplyr (version 1.1.3; [26]).

## 3. Results

A study sample comprising 627 individuals was examined, of which 209 (33.3%) were identified with orofacial clefts (OFC group), and 418 (66.7%) constituted the control group of randomly assigned healthy patients [aged 4 to 30 years (mean age 17.4 ± 13.6 years)]. Both groups exhibited identical gender distributions: women accounted for 43.54% of each group (OFC: 91; control: 182), and men comprised 56.46% (OFC: 118; control: 236). This gender parity ensures the comparability of the two cohorts in the analyses that follow. The analysis of the types of orofacial clefts (OFCs) in the OFC group reveals a predominant occurrence of unilateral cleft lip and palate (UCL/P), which affects over half of the cases (54.07%). Bilateral cleft lip and palate (BCL/P) and cleft palate only (CPO) are also present in substantial numbers (21.5%, 15.3% respectively), pointing to a diverse spectrum of cleft anomalies within the group.

Analyzing SNPs for potential determinants of orofacial cleft (OFC) incidence, we observed varying genotype distributions between the OFC and control groups across the studied SNPs (Table 2).

In the following analysis, we present a comprehensive evaluation of the potential associations between various SNP genotypes and the incidence of OFC, incorporating sex as a confounding variable (Table 3). Such adjustment affords a more precise estimation of impact by accounting for confounders across different strata, thereby enhancing the reliability of the assessment in a clinical context. This detailed analysis enhances our comprehension of the intricate genetic susceptibilities associated with OFC, supporting the development of specialized genetic screening protocols and elucidating the influence of sexual dimorphism on these predispositions.

Given that the stratum-specific ORs in Table 3 do not demonstrate statistically significant discrepancies, the Mantel–Haenszel adjusted OR serves as a suitable summary measure for elucidating the association between SNP genotypes and the incidence of OFC.

With an OR = 3.22, the rs7078 CC genotype has a significantly high odds ratio. Individuals identified with this genotype should receive priority in genetic screenings for OFC. The exposure effect on the outcome variable demonstrated homogeneity across all analyzed genotypes, indicating that the impact of genotypes on the incidence of OFC was consistent for both sexes. This uniformity in effect sizes across male and female groups suggests that the genetic influence on OFC risk is stable, irrespective of sex. This finding enhances the clinical understanding that the contribution of these genotypes to OFC risk is broadly applicable, supporting their inclusion in risk assessment models that do not need to differentiate based on sex.

Table 4 presents the regression coefficients of the multiple logistic regression models for OFC, and specifically for CL, CPO, and UCL/P.

For OFCs, the association of the rs7078 CC genotype was analyzed using multivariate models. The effect for this genotype maintained its statistical significance even with a diminished sample size that excluded cases with incomplete data on individual predictors. This suggests a strong and consistent association between thi genotype and OFC incidence, reinforcing its potential role as significant genetic markers.

The findings related to CL were less clear, especially in the multivariate analysis, because this condition is relatively rare. This lack of clarity in the results highlights the difficulty in achieving strong statistical power and emphasizes the need for further research with larger sample sizes to better understand the genetics of CL.

Significantly, the rs7078 CC genotype merits particular attention due to its consistent demonstration of significant associations, not only with OFCs in general but specifically with CPO and UCL/P. This recurrent identification highlights its potential importance in genetic predisposition to these specific subtypes of clefts. Additionally, this genotype again has shown considerable effects in analyses involving a multivariable approach, emphasizing its potential as a critical genetic marker in the clinical assessment and management of OFCs.

Table 5 presented below shows the strength of association between SNP genotypes and the incidence of specific cleft types (exclusively those genotypes that have demonstrated significant associations).

Based on the presented data in the above table, among the examined SNPs, four demonstrated significant associations, of which one genotype was identified as having protective properties against specific orofacial clefts (OFCs): rs7078 CT. Two genotypes were found to increase the odds ratio of OFC development: rs3769817 AA and rs7078 CC. The factor of sex does not significantly impact the association between genotype and the occurrence of specific cleft types. This finding reaffirms the predominant role of genetic factors in the pathogenesis of these conditions, transcending sex-related differences.

## 4. Discussion

In this study, we investigated the impact of four SNPs on the occurrence of clefts in the Polish population. Our analysis revealed that only the polymorphism rs7078 in the *MYH9* gene shows a significant correlation with the development of clefts, while the other three SNPs (rs3769817, rs1801131, rs13041247) do not have such an effect.

Individuals identified with this genotype should receive priority in genetic screenings for OFC. Early identification enables immediate initiation of intensive prenatal care, including frequent ultrasound checks aimed at early OFC detection and planning for necessary surgical interventions at or shortly after birth.

Numerous researchers have investigated the *MYH9* gene, particularly the SNP rs7078, in the context of orofacial cleft formation, mostly in Asian and American populations. Their studies have found a significant correlation between this polymorphism and the occurrence of orofacial clefts [13,27,28,29,30]. To date, no research has specifically examined this SNP within the Polish population; however, one investigation confirmed the influence of rs7078 among a Central European population [27]. Our study presents the first evidence linking rs7078 to cleft formation in this demographic, highlighting its potential role in genetic predisposition to these congenital anomalies among Poles.

The *MYH9* gene is responsible for encoding the protein non-muscle myosin heavy chain IIA (NMHC IIA), which is a crucial component of the cytoskeleton in non-muscle cells. This protein plays a significant role in various cellular processes. NMHC IIA is involved in maintaining cell shape and enabling cell movement through its role in the actin cytoskeleton. It interacts with actin filaments to generate contractile force, which is essential for cell motility, adhesion, and division. Moreover, during cell division, *MYH9* is crucial for cytokinesis, the final stage of mitosis where the cytoplasm of one cell divides to form two cells. The protein helps in forming the contractile ring that pinches the cell membrane to complete cell division. Furthermore, NMHC IIA is important in processes of wound healing, immune response, and tissue remodeling. It contributes to the dynamics of cell adhesion and migration by interacting with cell adhesion molecules. *MYH9* is involved in intracellular signaling pathways that regulate growth, differentiation, and the response to external stimuli [31,32]. In the context of OFCs, research suggests that variations in the *MYH9* gene, such as the SNP rs7078, might affect the gene’s function or expression, thereby influencing developmental processes critical for craniofacial formation. This makes *MYH9* a gene of interest in genetic studies of congenital anomalies like OFCs, regardless of ethnic origin. A high level of *MYH9* expression was detected specifically in the epithelial cells of the palatal shelves before their fusion. As the fusion process progressed, *MYH9* expression diminished and became confined to epithelial triangles, eventually disappearing completely once fusion was completed [33]. Based on our results and previous studies one must infer that rs7078 may play a significant role in the etiology of clefts worldwide.

The second studied SNP was rs1081131. This SNP is located in the gene MTHFR (methylenetetrahydrofolate reductase) and is known to affect the activity of the MTHFR enzyme, which plays a crucial role in folate metabolism [34,35]. Variants of rs1081131 have been associated with various health conditions, including colorectal cancer [35], cardiovascular disease [36], neural tube defects, and other disorders influenced by folate levels and methylation processes [37].

Although no association of rs1081131 with clefts was found in a North Indian population [38], in a Moroccan population there was a low association (OR = 0.24) [39]. On the other hand, the *MTHFR* rs1801131 gene polymorphism is strongly associated with OFCs among the Deutero Malay population in Indonesia [40]. Similar to our study, there were no significant differences in the frequencies of the *MTHFR* rs1081131 polymorphism in a Japanese cohort [41]. Therefore, the results for this SNP highly vary depending on the population, which may indicate that further studies are needed either in conjunction with other SNPs or in the Polish population, where this SNP may not have an impact.

*MAFB* (v-maf avian musculoaponeurotic fibrosarcoma oncogene homolog B) is a transcription factor belonging to the MAF (musculoaponeurotic fibrosarcoma) family of proteins. *MAFB* plays critical roles in gene regulation during embryonic development of many critical structures and organs, cell differentiation, and physiological processes [42]. First of all, *MAFB* is involved in the differentiation of neurons in the central nervous system [43]. Secondly, loss of *MAFB* function can lead to developmental defects in the kidneys and can lead to impairments in insulin production [44,45]. Thirdly, *MAFB* regulates the expression of genes responsible for shaping anatomical structures, such as bones and muscle tissues, and plays a role in controlling apoptosis and cell proliferation during embryogenesis [46]. A meta-analysis was conducted to examine the association between the rs13041247 and the risk of OFCs. The results suggested that there is an association in the overall pooled study population. However, this association was not significant in specific subpopulations, including East Asian and Caucasian groups. These studies encompassed a diverse range of populations, including East Asian, Caucasian, Indian, Mayan, Brazilian, and African cohorts [47]. But the Caucasian groups comprised populations only from Germany [48] and Brazil [49], what makes our study a significant contribution to the research on this SNP.

The last SNP we examined in our study was rs3769817 in the *SUMO1* gene (small ubiquitin-like modifier 1). It plays several crucial roles in embryogenesis, primarily through its involvement in the process of sumoylation, a post-translational modification that attaches SUMO moieties to target proteins. *SUMO1* is involved in regulation of the cell cycle and participates in DNA repair mechanisms. Moreover, *SUMO1* has been implicated in the development of craniofacial structures. Mutations in or dysregulation of *SUMO1* can lead to defects of facial morphogenesis [50]. The results of studies conducted on a large group of patients from Central Europe do not support the hypothesis that common or rare variants in *SUMO1* play a significant role in the development of OFCs in this ethnic group of patients [51], which is in accordance to our results. However, in a large cohort of Irish patients, the *SUMO1* SNP rs3769817 was associated with an increased risk of cleft palate (CP) (OR: 1.45; 95% CI: 1.06–1.99) for heterozygotes [52]. In a Chinese population, researchers investigated four different SNPs within the *SUMO1* gene. They observed an association with CLP for a specific combination of SNPs (haplotype), but none of the individual SNPs showed a significant association with the condition [53].

To sum up, our study focused on four SNPs and their association with orofacial clefts in the Polish population. We found that only *MYH9* rs7078 showed a significant correlation with OFC, highlighting its potential role in genetic predisposition among Central Europeans. In contrast, *MTHFR* rs1081131, *MAFB* rs13041247, and *SUMO1* rs3769817 did not show significant associations with CL/P in our study, aligning with findings in similar populations globally. These results underscore the need for further research to clarify the genetic factors contributing to orofacial cleft formation in different ethnic groups.

### Strengths and Limitations

The study’s strengths include a large sample size, enhancing statistical power and reliability. The homogeneity of the group, with participants from the same birthplace, minimizes geographic, environmental, and cultural variability. Ethnic uniformity reduces genetic differences, aiding clear result interpretation. Environmental consistency mitigates varying influences such as air pollution, healthcare access, and diet, ensuring more reliable outcomes. Uniform medical care allows for standardized diagnosis and treatment. The local focus aids in understanding regional risk factors and patterns. Participant clustering facilitates long-term observation and boosts cooperation, yielding higher data quality and retention.

On the other hand, many studies involving OFCs have yielded inconsistent results. We recognize that a study design focusing on the correlations among single genetic variants and various phenotypes may have limited power in detecting complex inheritance patterns, such as the synergistic involvement of different SNPs. Further research is needed to confirm the reported associations. Odds ratios (ORs) are often low to moderate when investigating the association of single genes with the risk of a complex trait likely influenced by many genes. This reflects the fact that specific phenotypes result from a combination of different genes (each contributing a small effect) and environmental factors. Therefore, it is essential to consider gene–environment interactions to explain the remaining genetic risk of non-syndromic orofacial clefts.

## 5. Conclusions

The research presented indicates a significant correlation between the rs7078 polymorphism and the occurrence of orofacial cleft palate in the Polish population, whereas the rs3769817, rs1801131, and rs13041247 SNPs do not show such a correlation.

## Figures and Tables

**Table 1:** Candidate genes and SNPs studied.

SNP	Gene	Genomic Position	Base Change	Location	MAF
rs7078	MYH9	Chr22:36281868	A/G	3 prime UTR variant	0.2766(G)
rs1081131	MTHFR	Chr1:11794419	T/G	exon	0.2277(G)
rs13041247	MAFB	Chr20:40640434	T/C	intron	0.3990(C)
rs3769817	SUMO1	Chr2:202214584	T/C	intron	0.8818(C)

**Table 2:** Genotype distributions of studied SNPs in OFC and control patients.

SNP with Genotypes	N	Group	
		OFC (*n* = 209)	Control (*n* = 418)
rs7078	569		
CC		24 (12.90%)	17 (4.44%)
CT		91 (48.92%)	200 (52.22%)
TT		71 (38.17%)	166 (43.34%)
rs10801131	519		
AA		84 (44.68%)	160 (48.34%)
AC		80 (42.55%)	138 (41.69%)
CC		24 (12.77%)	33 (9.97%)
rs13041247	558		
CC		19 (9.41%)	52 (14.61%)
CT		103 (50.99%)	160 (44.94%)
TT		80 (39.60%)	144 (40.45%)
rs3769817	505		
AA		2 (1.07%)	3 (0.94%)
AG		27 (14.44%)	63 (19.81%)
GG		158 (84.49%)	252 (79.25%)

**Table 3:** Estimation the measures of association strength between SNP genotypes and the incidence of OFC with confounding by sex (strata).

SNP	Genotype	OR with CI 95%	*p*_M-H adj OR = 1_	*p_Woolf homogeneity_*
rs7078	CC	3.22 (1.68–6.17)	**<0.001**	0.454
rs7078	CT	0.88 (0.62–1.24)	0.229	0.971
rs7078	TT	0.81 (0.56–1.15)	0.121	0.323
rs1081131	AA	0.86 (0.60–1.23)	0.203	0.969
rs1081131	AC	1.04 (0.72–1.49)	0.424	0.912
rs1081131	CC	1.32 (0.75–2.31)	0.168	0.990
rs13041247	CC	0.61 (0.35–1.06)	0.050	0.512
rs13041247	CT	1.27 (0.90–1.80)	0.085	0.323
rs13041247	TT	0.96 (0.68–1.37)	0.419	0.177
rs3769817	AA	1.16 (0.19–7.02)	0.435	0.528
rs3769817	AG	0.69 (0.42–1.12)	0.066	0.535
rs3769817	GG	1.42 (0.88–2.30)	0.076	0.718

**Table 4:** The regression coefficients of the multiple logistic regression models, with OFC (Nobs = 439), CL (Nobs = 419), CPO (Nobs = 487), and UCL/P (Nobs = 411) incidence as outcome variables.

Predictor	OFC	OR	CI 95%	*p*
(Intercept)	OFC Model 1	0.41	0.27–0.59	**<0.001**
rs7078 CC	OFC Model 1	2.52	1.18–5.41	**0.016**
(Intercept)	OFC Model 2	0.86	0.57–1.29	0.458
rs7078 CC	OFC Model 2	2.46	1.14–5.30	**0.021**
(Intercept)	CL	0.03	0.01–0.12	**<0.001**
rs3769817 AA	CL	11.32	0.50–115.00	0.054
(Intercept)	CPO	0.04	0.02–0.09	**<0.001**
rs7078 CC	CPO	3.19	1.04–8.89	**0.031**
rs7078 CT	CPO	0.66	0.25–1.64	0.378
(Intercept)	UCL/P Model 1	0.12	0.07–0.19	**<0.001**
rs7078 CC	UCL/P Model 1	2.33	0.98–5.20	**0.045**
(Intercept)	UCL/P Model 2	0.33	0.20–0.52	**<0.001**
rs7078 CC	UCL/P Model 2	2.31	0.98–5.20	**0.047**

**Table 5:** Estimation of the measures of association strength (significant only) between SNP genotypes and the incidence of specific cleft types, adjusted for sex confounding.

OFC	SNP	Genotype	OR with CI 95%	*p*_M-H adj OR = 1_
CL	rs3769817	AA	14.01 (1.35–145.24)	**0.002**
CPO	rs7078	CC	3.44 (1.29–9.16)	**0.005**
CPO	rs7078	CT	0.43 (0.19–0.95)	**0.017**
UCL/P	rs7078	CC	2.44 (1.21–4.92)	**0.005**

## Data Availability

All data are available from the corresponding author upon request.

## References

[1] Parker S.E., Mai C.T., Canfield M.A., Rickard R., Wang Y., Meyer R.E., Anderson P., Mason C.A., Collins J.S., Kirby R.S. (2010). Updated national birth prevalence estimates for selected birth defects in the United States, 2004–2006. Birth Defects Res. Part A Clin. Mol. Teratol..

[2] Dixon M.J., Marazita M.L., Beaty T.H., Murray J.C. (2011). Cleft lip and palate: Understanding genetic and environmental influences. Nat. Rev. Genet..

[3] Wehby G.L., Cassell C.H. (2010). The impact of orofacial clefts on quality of life and healthcare use and costs. Oral Dis..

[4] Compagnucci C., Martinus K., Griffin J., Depew M.J. (2021). Programmed Cell Death Not as Sledgehammer but as Chisel: Apoptosis in Normal and Abnormal Craniofacial Patterning and Development. Front. Cell Dev. Biol..

[5] Payer D., Krimmel M., Reinert S., Koos B., Weise H., Weise C. (2022). Oral healthrelated quality of life in patients with cleft lip and/or palate or Robin sequence. J. Orofac. Orthop..

[6] Zawiślak A., Wędrychowska-Szulc B., Grocholewicz K., Janiszewska-Olszowska J. (2023). Craniofacial Cephalometric Morphology in Caucasian Adult Patients with Cleft Palate Only (CPO). Diagnostics.

[7] Zawiślak A., Woźniak K., Kawala B., Gupta S., Znamirowska-Bajowska A., Janiszewska-Olszowska J., Lubiński J., Calvo-Guirado J.L., Grocholewicz K., Jakubowska A. (2023). *IRF6* and *FGF1* polymorphisms in non-syndromic cleft lip with or without cleft palate in the Polish population. Open Med..

[8] Babai A., Irving M. (2023). Orofacial Clefts: Genetics of Cleft Lip and Palate. Genes.

[9] Saleem K., Zaib T., Sun W., Fu S. (2019). Assessment of candidate genes and genetic heterogeneity in human non syndromic orofacial clefts specifically non syndromic cleft lip with or without palate. Heliyon.

[10] Sun J., Li M., Sun H., Lin Z., Shi B., Jia Z. (2024). Genetic association and functional validation of ZFP36L2 in non-syndromic orofacial cleft subtypes. J. Hum. Genet..

[11] Zhu M., Zhao S. (2007). Candidate Gene Identification Approach: Progress and Challenges. Int. J. Biol. Sci..

[12] Reynolds K., Zhang S., Sun B., Garland M.A., Ji Y., Zhou C.J. (2020). Genetics and signaling mechanisms of orofacial clefts. Birth Defects Res..

[13] Wang Y., Li D., Xu Y., Ma L., Lu Y., Wang Z., Wang L., Zhang W., Pan Y. (2018). Functional Effects of SNPs in MYH9 and Risks of Nonsyndromic Orofacial Clefts. J. Dent. Res..

[14] Phan H.D.B., Phuong L.H., Vu H.A. (2022). Association of Single-Nucleotide Polymorphisms of *MAFB* Gene with Nonsyndromic Cleft Lip with or without Cleft Palate in Kinh Vietnamese Patients. Indian J. Plast. Surg..

[15] WHO (2019). International Statistical Classification of Diseases and Related Health Problems 10th Revision. https://icd.who.int/browse10/2019/en.

[16] Lahiri D.K., Schnabel B. (1993). DNA isolation by a rapid method from human blood samples: Effects of MgCl2, EDTA, storage time, and temperature on DNA yield and quality. Biochem Genet..

[17] Zawiślak A., Woźniak K., Agirre X., Gupta S., Kawala B., Znamirowska-Bajowska A., Grocholewicz K., Lubiński J., Prosper F., Jakubowska A. (2021). Association of ABCA4 Gene Polymorphisms with Cleft Lip with or without Cleft Palate in the Polish Population. Int. J. Environ. Res. Public Health.

[18] Wald A. (1943). Tests of statistical hypotheses concerning several parameters when the number of observations is large. Trans. Am. Math. Soc..

[19] Jewell N.P. (2004). Statistics for Epidemiology.

[20] R Core Team (2023). R: A Language and Environment for Statistical Computing.

[21] Lüdecke D. (2023). sjPlot: Data Visualization for Statistics in Social Science. R Package Version 2.8.15. https://CRAN.R-project.org/package=sjPlot.

[22] Lüdecke D., Ben-Shachar M., Patil I., Waggoner P., Makowski D. (2021). Performance: An R Package for Assessment, Comparison and Testing of Statistical Models. J. Open Source Softw..

[23] Makowski D., Lüdecke D., Patil I., Thériault R., Ben-Shachar M., Wiernik B. (2023). Automated Results Reporting as a Practical Tool to Improve Reproducibility and Methodological Best Practices Adoption. CRAN. https://easystats.github.io/report/.

[24] Sjoberg D., Whiting K., Curry M., Lavery J., Larmarange J. (2021). Reproducible Summary Tables with the gtsummary Package. R J..

[25] Stevenson M., Sergeant E. (2024). epiR: Tools for the Analysis of Epidemiological Data. R Package Version 2.0.75. https://CRAN.R-project.org/package=epiR.

[26] Wickham H., François R., Henry L., Müller K., Vaughan D. (2023). dplyr: A Grammar of Data Manipulation. R Package Version 1.1.3. https://CRAN.R-project.org/package=dplyr.

[27] Birnbaum S., Reutter H., Mende M., de Assis N.A., Diaz-Lacava A., Herms S., Scheer M., Lauster C., Braumann B., Schmidt G. (2009). Further evidence for the involvement of MYH9 in the etiology of non-syndromic cleft lip with or without cleft palate. Eur. J. Oral Sci..

[28] Chiquet B.T., Hashmi S.S., Henry R., Burt A., Mulliken J.B., Stal S., Bray M., Blanton S.H., Hecht J.T. (2009). Genomic screening identifies novel linkages and provides further evidence for a role of MYH9 in nonsyndromic cleft lip and palate. Eur. J. Hum. Genet..

[29] Jia Z.L., Li Y., Chen C.H., Li S., Wang Y., Zheng Q., Shi B. (2010). Association among polymorphisms at MYH9, environmental factors, and nonsyndromic orofacial clefts in western China. DNA Cell Biol..

[30] Peng H.H., Chang N.C., Chen K.T., Lu J.J., Chang P.Y., Chang S.C., Wu-Chou Y.H., Chou Y.T., Phang W., Cheng P.J. (2016). Nonsynonymous variants in MYH9 and ABCA4 are the most frequent risk loci associated with nonsyndromic orofacial cleft in Taiwanese population. BMC Med. Genet..

[31] Pecci A., Ma X., Savoia A., Adelstein R.S. (2018). MYH9: Structure, functions and role of non-muscle myosin IIA in human disease. Gene.

[32] An Q., Dong Y., Cao Y., Pan X., Xue Y., Zhou Y., Zhang Y., Ma F. (2022). *Myh9* Plays an Essential Role in the Survival and Maintenance of Hematopoietic Stem/Progenitor Cells. Cells.

[33] Martinelli M., Di Stazio M., Scapoli L., Marchesini J., Di Bari F., Pezzetti F., Carinci F., Palmieri A., Carinci P., Savoia A. (2007). Cleft lip with or without cleft palate: Implication of the heavy chain of non-muscle myosin IIA. J. Med. Genet..

[34] Aarabi M., San Gabriel M.C., Chan D., Behan N.A., Caron M., Pastinen T., Bourque G., MacFarlane A.J., Zini A., Trasler J. (2015). High-dose folic acid supplementation alters the human sperm methylome and is influenced by the MTHFR C677T polymorphism. Hum. Mol. Genet..

[35] Wiik M.U., Negline M., Beisvåg V., Clapham M., Holliday E., Dueñas N., Brunet J., Pineda M., Bonifaci N., Aretz S. (2023). MTHFR C677T and A1298C polymorphism’s effect on risk of colorectal cancer in Lynch syndrome. Sci. Rep..

[36] Raghubeer S., Matsha T.E. (2021). Methylenetetrahydrofolate (MTHFR), the One-Carbon Cycle, and Cardiovascular Risks. Nutrients.

[37] Aaa Leung K.Y., Pai Y.J., Chen Q., Santos C., Calvani E., Sudiwala S., Savery D., Ralser M., Gross S.S., Copp A.J. (2017). Partitioning of One-Carbon Units in Folate and Methionine Metabolism Is Essential for Neural Tube Closure. Cell Rep..

[38] Avasthi K.K., Agarwal A., Agarwal S. (2022). Association of *MTHFR, BMP4, TGFA* and *IRF6* Polymorphisms with Non-Syndromic Cleft lip and Palate in North Indian Patients. Avicenna J. Med. Biotechnol..

[39] Rafik A., Rachad L., Kone A.S., Nadifi S. (2019). MTHFR C677T polymorphism and risk of nonsyndromic cleft lip with or without cleft palate in the Moroccan population. Appl. Clin. Genet..

[40] Nasroen S.L., Tammama T., Darwis R.S., Adil A., Rahmutia S., Maskoen A.M., Gani B.A. (2023). The *IRF6* rs2013162 and *MTHFR* A1298C rs1801131 Gene Polymorphisms Related to non-Syndromic Cleft lip and Palate among Deutero-Malay in Indonesia. Cleft Palate Craniofacial J..

[41] Komiyama Y., Koshiji C., Yoshida W., Natsume N., Kawamata H. (2020). 5,10-Methylenetetrahydrofolate reductase (*MTHFR*) C677T/A1298C polymorphisms in patients with nonsyndromic cleft lip and palate. Biomed. Rep..

[42] Fujino M., Ojima M., Takahashi S. (2023). Exploring Large MAF Transcription Factors: Functions, Pathology, and Mouse Models with Point Mutations. Genes.

[43] Pai E.L., Chen J., Fazel Darbandi S., Cho F.S., Chen J., Lindtner S., Chu J.S., Paz J.T., Vogt D., Paredes M.F. (2020). *Maf* and *Mafb* control mouse pallial interneuron fate and maturation through neuropsychiatric disease gene regulation. eLife.

[44] Moriguchi T., Hamada M., Morito N., Terunuma T., Hasegawa K., Zhang C., Yokomizo T., Esaki R., Kuroda E., Yoh K. (2006). MafB is essential for renal development and F4/80 expression in macrophages. Mol. Cell. Biol..

[45] Xiafukaiti G., Maimaiti S., Ogata K., Kuno A., Kudo T., Shawki H.H., Oishi H., Takahashi S. (2019). MafB Is Important for Pancreatic β-Cell Maintenance under a MafA-Deficient Condition. Mol. Cell. Biol..

[46] McGonnell I.M., McKay I.J., Graham A. (2001). A population of caudally migrating cranial neural crest cells: Functional and evolutionary implications. Dev. Biol..

[47] Huang L., Liang X., Ou Y., Tang S., He Y. (2020). Association between 20q12 rs13041247 polymorphism and risk of nonsyndromic cleft lip with or without cleft palate: A meta-analysis. BMC Oral Health.

[48] Reiter R., Brosch S., Goebel I., Ludwig K.U., Pickhard A., Hoegel J., Schloemer G., Mangold E., Kubisch C., Borck G. (2015). A post GWAS association study of SNPs associated with cleft lip with or without cleft palate in submucous cleft palate. Am. J. Med. Genet. Part A.

[49] Fontoura C., Silva R.M., Granjeiro J.M., Letra A. (2012). Further evidence of association of the ABCA4 gene with cleft lip/palate. Eur. J. Oral Sci..

[50] Lee A., Zhu Y., Sabo Y., Goff S.P. (2019). Embryonic Cells Redistribute SUMO1 upon Forced SUMO1 Overexpression. mBio.

[51] De Assis N.A., Nowak S., Ludwig K.U., Reutter H., Vollmer J., Heilmann S., Kluck N., Lauster C., Braumann B., Reich R.H. (2011). SUMO1 as a candidate gene for non-syndromic cleft lip with or without cleft palate: No evidence for the involvement of common or rare variants in Central European patients. Int. J. Pediatr. Otorhinolaryngol..

[52] Carter T.C., Molloy A.M., Pangilinan F., Troendle J.F., Kirke P.N., Conley M.R., Orr D.J., Earley M., McKiernan E., Lynn E.C. (2010). Testing reported associations of genetic risk factors for oral clefts in a large Irish study population. Birth Defects Res. Part A Clin. Mol. Teratol..

[53] Song T., Li G., Jing G., Jiao X., Shi J., Zhang B., Wang L., Ye X., Cao F. (2008). SUMO1 polymorphisms are associated with non-syndromic cleft lip with or without cleft palate. Biochem. Biophys. Res. Commun..

